# Inducing ferroptosis to improve cancer therapy: a promising tool for enhancing immunotherapy

**DOI:** 10.1186/s13046-025-03593-3

**Published:** 2025-12-03

**Authors:** Matteo Caforio, Stefano Iacovelli, Franco Locatelli, Valentina Folgiero

**Affiliations:** 1 Department of Onco-Hematology and Cell and Gene Therapy, Bambino Gesù Children’s Hospital, IRCCS, Rome, Italy; 2 Department of Life Sciences and Public Health, Catholic University of the Sacred Heart, Rome, Italy

**Keywords:** Ferroptosis, Cancer, Immunotherapy

## Abstract

**Background:**

The discovery of ferroptosis as a novel mechanism of cell death has opened the door to a new scenario in which it could be used to support current cancer therapy, particularly in cases of relapse. Several compounds have been developed aimed to inhibit or induce ferroptosis in cancer cells by acting on different signaling pathways caable of activating or repressing, respectively, this cell death mechanism.

**Main body:**

This review shows how treatmenting cancer cells with ferroptosis inducers results in improved efficacy of immunotherapy. Indeed, the advantage of affecting ferroptosis lies in the capacity of compounds to improve immune system compartments. The involvement of ferroptosis in cancer treatment is now emerging, demonstrating the high translational potential of this approach capable of carrying out an immune response against tumors, dendritic cells (DC), regulatory T cells (Treg), Natural Killer cells (NK) and tumor-associated macrophages (TAM) exert an interesting role. Some immune check-point inhibitors (ICIs) have been approved as cancer immunotherapy, because they target cytotoxic T lymphocyte-associated antigen 4 (CTLA4), programmed cell death protein 1 (PD-1) and its ligand PD-L1. For this reason, promising results have been achieved by combining ferroptosis inducers with ICIs. At the same time, combining Chimeric Antigen Receptor (CAR) T-cell therapy with ferroptosis inducers shows promising anti-tumor activity, particularly in solid tumors. This approach demonstrates how the modulation of ferroptosis may improve the efficacy of CAR T-cells treatment by promoting tumor cell death and enhancing immunogenicity.

**Conclusion:**

In conclusion the development of clinical trials aimed at testing the efficacy of ferroptosis induction in combination with current cancer therapy will be the definitive proof of the valid opportunity provided by this therapeutic approach.

## Background

Once almost all regulated cell death pathways, in mammalian cells, depended on the activation of caspase-dependent apoptosis [1, 2]. More recent is the discovery of several regulated non-apoptotic cell death pathways activated in specific disease states, including poly (ADP-ribose) polymerase-1 (PARP-1) and apoptosis inducing factor 1 (AIF1)-dependent cell-death, caspase-1-dependent pyroptosis and receptor interacting protein kinase 1 (RIPK1) dependent necroptosis [3–5]. It has been hypothesized that additional regulated forms of non-apoptotic cell death likely remain to be discovered that mediate cell death in other developmental or pathological circumstances.

A promising candidate is ferroptosis, a form of regulated cell death, driven by lipid peroxidation and shaped by metabolic and antioxidant pathways.

It is characterized by metabolic dysfunction that leads to the production of cytosolic reactive oxygen species (ROS), such as hydrogen peroxide (H_2_O_2_), and lipid peroxidation and shares no morphological, bioenergetic or other similarities with apoptotic or necrotic death, or with autophagy [6].

Ferroptosis is characterized by intracellular Fe^2+^ accumulation, which drives lipid peroxidation and ROS production [7]. It can be triggered by a wide range of physiological conditions and pathological stresses, which can be categorized into exogenous pathways (relying on various transport proteins) and endogenous pathways (involving the inhibition of intracellular antioxidant enzymes) [8]. Furthermore, the regulatory mechanisms governing ferroptosis exhibit significant divergence across cell types and conditions. These regulatory mechanisms encompass the Glutathione Peroxidase 4 (GPX4) pathway, iron metabolism-related processes, and lipid metabolism involved pathways [9]. The discovery of ferroptosis emerged from the identification in 2003 of small molecules that induced a non-apoptotic form of cell death [10]: two novel oncogenic RAS Selective Lethal (RSL) small molecules named eradicator of Ras and ST (Erastin) and Ras Selective Lethal 3 (RSL3) [10, 11] directed to the RAS family of small GTPases (HRAS, NRAS and KRAS), commonly mutated in cancer [12]. These two compounds do not induce morphological changes or biochemical processes like apoptosis, such as chromatin margination or cleavage of PARP [10]. Otherwise by Erastin, it is observed a unique ‘dysmorphic’ mitochondrial phenotype and induction of a unique set of genes compared to cell death or cytostasis triggered by pro-apoptotic or pro-necrotic agents [6]. Analysis of the Erastin mechanism of action provided the first insight into the proteins and pathways necessary to prevent the onset of ferroptosis identifying inhibition of the cystine (Cys2)/glutamate(Glu) antiporter, termed system xCT, as the key mechanism of action [6, 13]. The system xCT is a heterodimeric cell surface amino acid antiporter composed of the twelve-pass transmembrane transporter protein SLC7A11 (xCT) linked by a disulfide bridge to the single-pass transmembrane regulatory protein SLC3A2 [14]. The xCT system imports extracellular Cys2 in exchange for intracellular Glu a process (Cysteine (Cys)-dependent Glutathione (GSH) synthesis) that normally antagonizes L-ROS accumulation.

Through the study of ferroptosis inducer, RSL3, it is now evident that GPX4 is its target. This enzyme is a GSH-dependent enzyme that reduces lipid hydroperoxides (L-OOH) to lipid alcohols (L-OH). GPX4, therefore, normally limits the iron-dependent formation of highly reactive lipid alkoxy radicals (L-O·) from L-OOH [15, 16] and its inhibition leads to the rapid accumulation of L-ROS and cell death [15, 17]. In response to inhibition of the xCT system or GPX4 inactivation, ferroptotic cell death involves the iron-dependent accumulation of L-ROS and depletion of polyunsaturated fatty acids (PUFAs) [18, 19].

L-ROS are typically formed from PUFA chains of membrane lipids. PUFAs are susceptible to both enzymatic (e.g., lipoxygenase-catalyzed) and non-enzymatic (e.g., ROS-catalyzed) oxidation, leading to the formation of lipid hydroperoxides (L-OOH). In the presence of iron, L-OOH can form toxic lipid radicals such as the L-O alkoxy radical. These lipid radicals can extract protons from adjacent PUFAs, initiating a new round of lipid oxidation and further propagation of oxidative damage from one lipid to another [20]. PUFA oxidation and free-radical-mediated damage can ultimately result in PUFA fragmentation into a variety of products [20]. The detoxification of reactive lipid intermediate 4-hydroxynonenal (4-HNE) can be formed downstream of oxidative PUFA fragmentation. Even if the involvement of the iron in the execution of ferroptosis is essential, it is still unclear how it promotes the mechanism. Probably the presence of iron chelators prevents iron from donating electrons to oxygen to form ROS [21] preventing ferroptosis.

### Ferroptosis and epigenetics

Epigenetic modifications such as histone post-translational modifications (PTMs), DNA and RNA methylation, and non-coding RNA (ncRNA) regulation can exert a role in regulating tumor ferroptosis [22]. PTMs are a group of multifunctional epigenetic marks that regulate the conformation of chromatin and the accessibility of transcription factors, co-activators and co-inhibitors as well as elements involved in transcription, DNA damage, apoptosis, and cell cycle regulation [23, 24].

#### Histone methylation

**Fig. 1:**
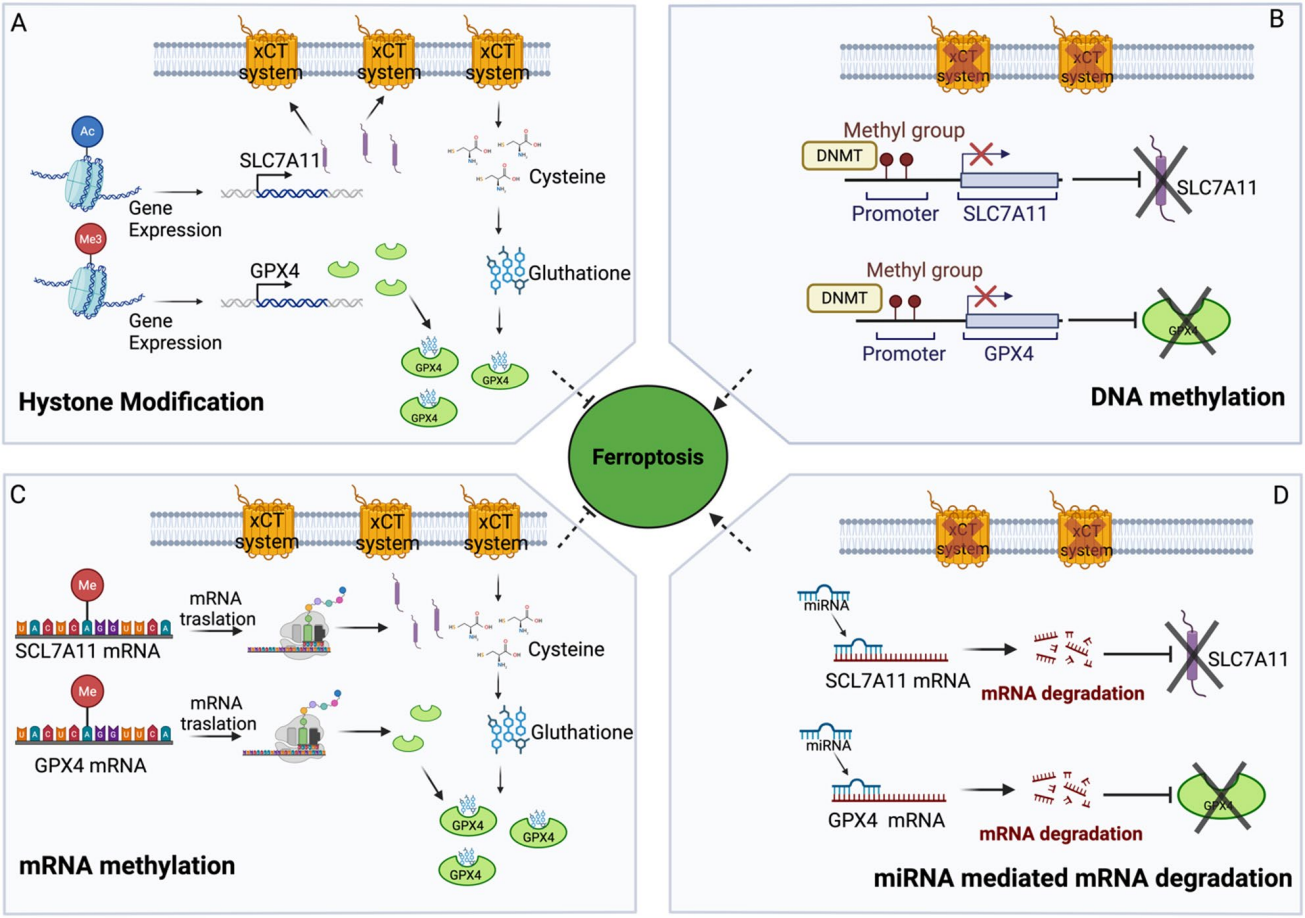
Description of different types of epigenetic modifications that control ferroptotic mechanisms. **A** Chromatin modification involves hystone acetylation, that can promote SLC7A11 expression, and hystone tri-methylation, that can promote GPX4 expression. Under these conditions the cell is protected from ferroptosis. **B** DNA Methylation of the promoter region of SLC7A11 and GPX4 induces the repression of their expression. In this situation the cells are more susceptible to ferroptosis mechanisms. **C** Methylation of adenine at position 6 of the mRNA is associated with an elevated translation mechanism. Methylation in SLC7A11 and GPX4 mRNAs induces their expression and inhibits ferroptosis. **D** miRNA can target and destroy mRNA of SLC7A11 and GPX4. This condition is able to favor ferroptosis mechanism

The abundance of GPX4 in tumor cells can be related to its methylated level due to increased levels of histone methylation in H3K4me3 on its promoter that results in the inhibition of ferroptosis [25]. The inhibition of GPX4 transcription can be due to methylation of EGR1 that directly binds GPX4 promoter [26]. The same methylation can be increased on ACSL3 promoter inhibiting ferroptosis [27] (Fig. 1A).

#### Histone acetylation

Histone acetylation, directed by histone acetyltransferase (HAT) and histone deacetylases (HDAC), belonging to the Bromodomain-containing protein, leads to nucleosome disaggregation and gene transcriptional activation. High H3K27ac levels improves the recognition of acetylation sites on GPX4 and SLC7A11 genomic protein affecting ferroptosis [28] (Fig. 1A).

#### DNA methylation

This process is often inversely linked to genes expression levels and in the context of ferroptosis it influences lipid metabolism. DNA methylation can directly regulate the expression of ferroptosis-related molecules such as GPX4 and SLC7A11 [29, 30]. Indeed, methylation of GPX4 promoter catalyzed by DNMT1, DNMT3A and DNMT3B induces ferroptosis and viceversa the use of DNMT1 inhibitor induces ferroptosis in gastric cancer by indirect inactivation of the xCT system (Fig. 1B).

#### RNA methylation

RNA methylation stands as a prominent area in epigenetic investigations, constituting over 60% of all RNA modifications, with RNA N6-methyladenine (m6A) emerging as the most prevalent mRNA post-transcriptional alteration [31]. In hepatoblastoma and lung adenocarcinoma ferroptosis is inhibited by enhancement of SCL7A11 mRNA stability by the m6A RNA guide, METTL3 [32] (Fig. 1C).

#### NcRNA

MicroRNAs (miRNA), non-coding single-stranded RNA made of about 22 nucleotides, regulate gene expression at the mRNA level by binding to the 3’UTR to inhibit mRNA translation or promote mRNA degradation. SCL7A11 can be downmodulated by miRNAs to induce ferroptosis, while GPX4 can be inhibited to promote ferroptosis [33–38]. Furhtermore, the abnormal expression of long non-coding RNAs (lncRNA) is related to tumors by their involvement in controlling ferroptosis through post-transcriptional process. It has been demonstrated that lncRNA can upregulate SCL7A11 expression to inhibit ferroptosis [39] (Fig. 1D).

#### Ubiquitination

The complex of enzymes involved in ubiquitination (E1, E2 and E3) utilizing ATP as source of energy, is aimed at protein regulation in collaboration with the complementary deubiquitination process driven by DUBs enzymes. Recent studies have focused on the role of protein degradation through ubiquitination under condition of iron overload in order to investigate the effect produced on ferroptosis [40]. SCL7A11 and GPX4 proteins are the pivotal target of E3 members of Tripartite motifs (TRIM) family resulting in induction of ferroptosis. In particular GPX4 can be ubiquitinated through the interaction with TRIM46 [41] while SCL7A11 is degraded by TRIM 26 [42]. Also, Ferroptosis suppressor protein 1 (FSP1) is a target of a member of TRIM family through the TRIM54-mediated ubiquitination resulting in the induction of ferroptosis in hepatocellular carcinoma [42].

#### Ubiquitin-like modification

Among the ubiquitin-like modification occurring through the covalent conjugation of ubiquitin fold modifier protein 1 (UFM1) to specific lysine residue, SUMOylation acts through the attachment to epsilon-amino groups of lysine residues within target proteins in order to degrade them [43, 44]. The enzymatic reactions during SUMOylation are involved in the control of ferroptosis through the regulation of several proteins like SLC7A11 or FSP1 [45, 46].

### Ferroptosis and cancer

#### GPX4 pathway

**Fig. 2:**
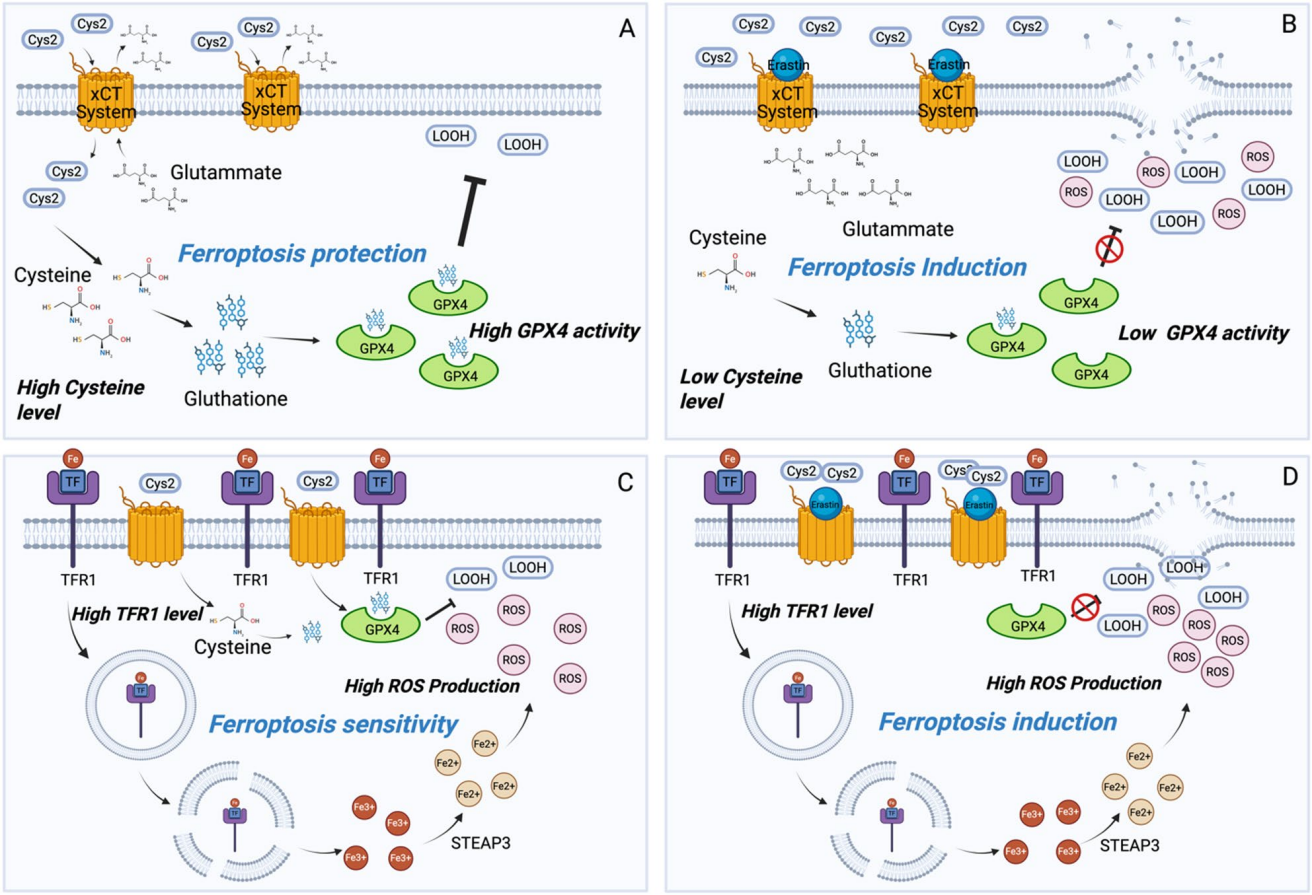
Ferroptosis pathways in cancer cells: **A** Many cancer cells exhibit high SLC7A11 and GPX4 expression. SLC7A11 increases the intracellular level of cys2 which is suddenly cleaved in two cys. Cys is important for the formation of GSH, an essential molecule for GPX4 function, that shows high activity in cancer cell and protects against ferroptosis. **B** When cancer cells are treated with SLC7A11 inhibitor (Erastin), cystein level is reduced and cancer cells are not protected from ferroptosis that, in this condition, is induced. **C** The interaction between TFR1 and its ligand (TF), induces the internalization and the next release of Iron into cells. High TFR1 expression in cancer favors the Iron-dependent-ROS production that, in turn, promotes lipid peroxidation and ferroptosis sensitivity. **D** Induction of ferroptosis in cancer cells with high TFR1 expression induces high ferroptotic effect and can be used as a combined therapy

Precise modulation of the GPX4-centered signaling pathway represents a core intervention point and a pivotal therapeutic target for the accurate manipulation and regulation of ferroptosis initiation and progression and constitutes a cardinal determinant governing neoplastic cell death. Modulation of GPX4 expression levels, encompassing both transcriptional upregulation and downregulation, exerts a demonstrably significant influence on the vulnerability of neoplastic cells to ferroptosis-inducing agents, providing a promising trajectory for the innovative design and development of targeted therapeutics in oncological intervention. The xCT system transporter, through which GPX4 enzyme executes its canonical catalytic activity, is a heterodimeric proteinaceous complex, that is functionally and structurally constituted by two obligate subunits, SLC7A11 and SLC3A2, which operate in a tightly coordinated and synergistic manner. Erastin, glutamic acid, sorafenib, and sulfasalazine, among others, achieve ferroptosis induction through the specific inhibition of the xCT system transporter functionality. This targeted inhibition effectively impedes the cellular uptake and influx of extracellular Cys2, thereby indirectly suppressing GPX4 enzymatic activity and ultimately leading to the highly efficient induction of ferroptosis in tumor cells. In parallel, a distinct class of compounds, encompassing RSL3, ML162, FIN56, and FINO2, adopt an alternative yet convergent strategy by directly targeting the GPX4 protein molecule, exerting direct enzymatic inhibitory effects [47] (Fig. 2A, B).

#### Iron metabolism

Intracellular iron levels are precisely regulated and mainly depend on the absorption, storage, and discharge of iron. Iron dietary intake is the primary source, a fraction is stored in red blood cells as a reserve, while in peripheral tissues it is stored in the form of ferritin and iron-sulfur clusters. Iron transport from extracellular to the intracellular space is mediated by Transferrin (TF) and Transferrin Receptor 1 (TFR1) at the transcriptional level [48]. TF is a 76-kDa glycoprotein that reversibly binds to iron in a pH-dependent manner. Iron efficiently binds to TF outside cells at pH ∼7.4 and iron is released from TF when delivered to acidic endosomes (pH ∼5.5) by receptor mediated endocytosis. It has been demonstrated that TF is essential for amino acid starvation-induced ferroptosis [49] suggesting its role as key positive regulator of ferroptotic cell death. TFR1 is a dimeric glycoprotein receptor at the surface of plasma cells. Following binding to TFR1, the TF-TFR1 complex is internalized via receptor-mediated endocytosis. The acidic environment of endosome induces the release of iron that needs to be reduced from Fe^3+^ to Fe^2+^ by transmembrane ferrireductase STEAP3. Then TFR1 and TF are recycled back to the cell surface and extracellular fluid, respectively [50]. The increased expression of TFR1 in malignant cells is aimed mainly at meeting the high requirement of iron for cell proliferation. However, tumor cells which presented high amount of TFR1 were more sensitive to cysteine deprivation-induced ferroptosis. According to this, TFR1 overexpression enhances ferroptosis, whereas TFR1 inhibition decreases ferroptosis level, suggesting a key role of TFR1/TF system in this process [51]. Therefore, inducing ferroptosis in TFR1-expressing tumors is a potential cancer treatment strategy (Fig. 2C, D).

#### FSP1 pathway

**Fig. 3:**
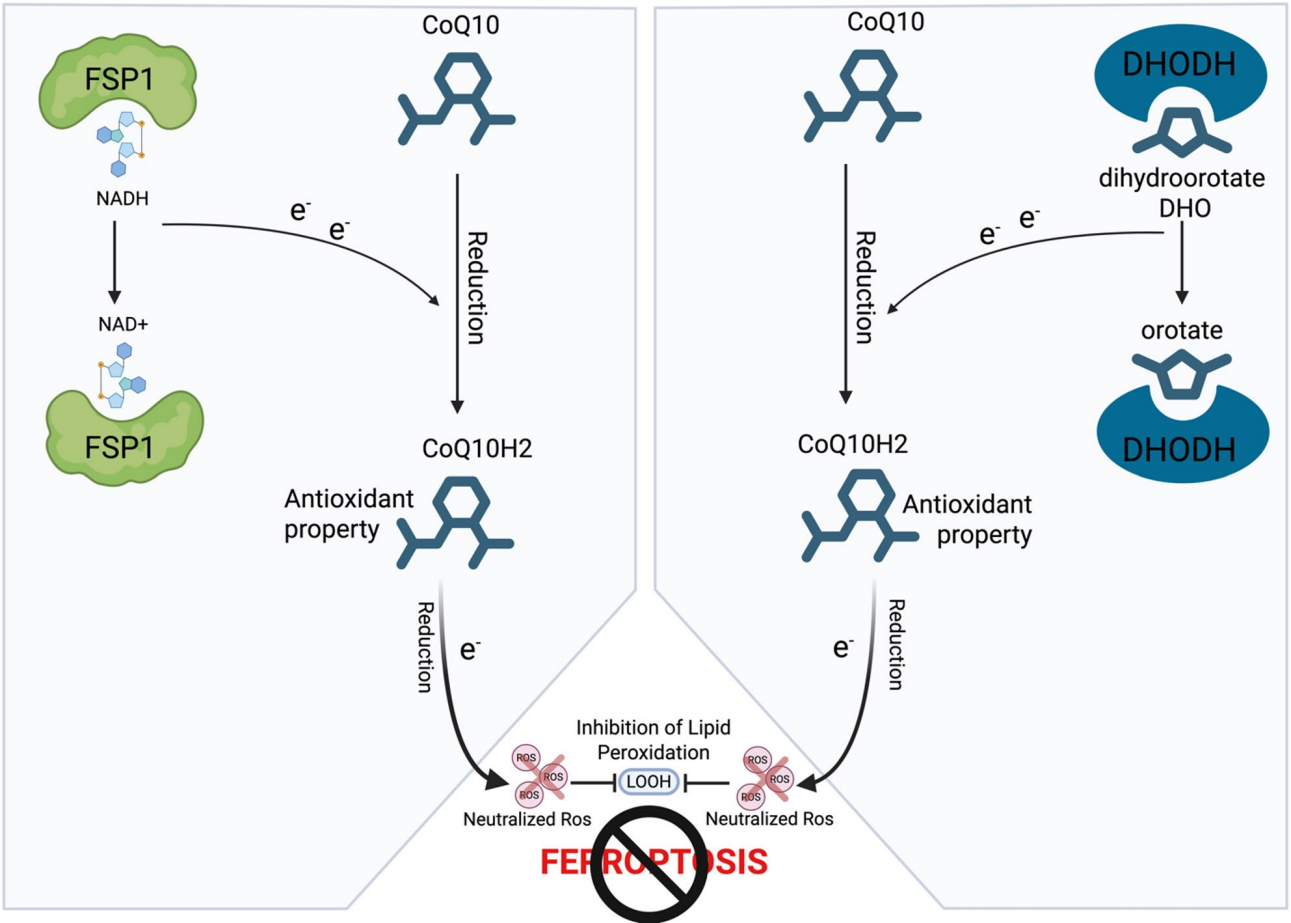
FSP-1 and DHODH role in ferroptosis: FSP-1(left) and (DHODH (right) act trough the consume of NADH/NADPH as an NADH/NADPH-dependent CoQ10 oxidoreductase to reduce CoQ10 (also called ubiquinone) to CoQ10H2 (ubiquinol) in order to inhibit ferroptosis. Electrons are transferred to ubiquinone (CoQ10), capable of capturing antioxidants and inhibiting lipid peroxide formation resulting in neutralization of Ros

FSP1 functions through the FSP1-coenzyme Q10, (CoQ10)-NAD(P)H axis and the vitamin K redox cycle controlling a redistribution of mitochondrial membrane potential and intracellular Ca2 + levels [52]. FSP1, acting in parallel with GPX4, represents another major regulator of ferroptosis [53]. In particular, its mechanism of action concerns the consume of NADH/NADPH as an NADH/NADPH-dependent CoQ10 oxidoreductase to reduce CoQ10 (also called ubiquinone) to CoQ10H2 (ubiquinol) (Fig. 3 left). CoQ10 is localized in the inner membrane of mitochondria while CoQ10H2 is an antioxidant able to capture free radicals to prevent lipid peroxidation in the cell membrane, thus inhibiting ferroptosis in tumor cells [54]. Since the regulation of ferroptosis and its target proteins represents a promising strategy for cancer treatment, targeting FSP1 to weaken its ability to inhibit ferroptosis may be a viable approach. Multiple FSP1 inhibitors have been developed, including iFSP1, icFSP1, viFSP1, and FSEN1 [55–58]. icFSP1 and FSEN1 have adequate pharmacokinetics in mice and have shown efficacy in vivo [56, 57, 59].

#### DHODH

Dihydroorotate dehydrogenase (DHODH) is an iron-containing flavin-dependent enzyme which plays a crucial role in the *de novo* synthesis of pyrimidine [60]. It is located in the inner membrane of mitochondria, where it catalyzes the conversion of dihydroorotate to orotate in a redox reaction, which is the fourth of six universally conserved enzymatic reactions in the pyrimidine de novo synthetic pathway [61, 62].

During synthesis, DHODH uses CoQ10 as coenzyme to perform the catalysis, converting CoQ10 to CoQ10H2. In this reaction electrons are transferred to ubiquinone (CoQ10), which can capture antioxidants and inhibit lipid peroxide formation [63]. Thus, DHODH inactivation results in widespread lipid peroxide accumulation and mitochondrial ferroptosis [64, 65] (Fig. 3. Right). DHODH might play an important role in cancer development and progression of cancer due to a severe proliferation defect associated with its deficiency [66]. In addition, it is really recent the demonstration of its role in c-Myc-driven tumors. DHODH interaction impedes SKP2-dependent c-MYC degradation and at the same time c-Myc transcriptionally activates DHODH sustaining anti-ferroptotic activity [67].

Many DHODH inhibitors with different structures have been reported [68], such as canonical DHODH inhibitors BRQ, leflunomide [69], teriflunomide [70], ALASN003 [61], BAY2202234 [71].

### Ferroptosis effect on tumor microenvironment

Cancer immunotherapy, highly developed and used for cancer treatments in the last years, exerts its mechanism enhancing the activation of the immune system in the tumor microenvironment (TME) to target and kill malignant cells [72, 73]. Since it shows favorable anti-tumor efficacy, it has become a primary choice in the clinical management of various solid tumors, including melanoma [74] pancreatic carcinoma, mammary carcinoma, ovarian carcinoma, and non-small cell lung carcinoma [75–79]. Preliminary evidence was produced by inhibitors capable of blocking the interaction between immune cells and cancer cells like PD-1, expressed by T-cells, and PDL1 expressed by cancer cells with the aim of rescuing T-cells anti-tumor activity [80]. More recent is the development of engineered immune system cells capable of restoring their anti-tumor activity in autologous and allogenic transplantation as Chimeric Antigen Receptor (CAR) T-cells or NK cells [81]. Despite the success of these examples of Advanced Therapy Medicinal Products (ATMP)s, cases of tumor relapse still occur prompting the development of combined approaches. For this reason the significance of the interplay between ferroptosis and immunotherapy in cancer treatment is becoming increasingly recognized [47].

Recent emerging evidence shows that ferroptosis plays an important role in regulation of the functions of cells of immune system [82]. This concept is strictly linked to the TME, as the tumor microenvironment recruits many immune cells to counteract tumor growth through the activity of specific cells crucial for influencing tumor proliferation.

#### Ferroptosis-sensitive immune cells

**Fig. 4:**
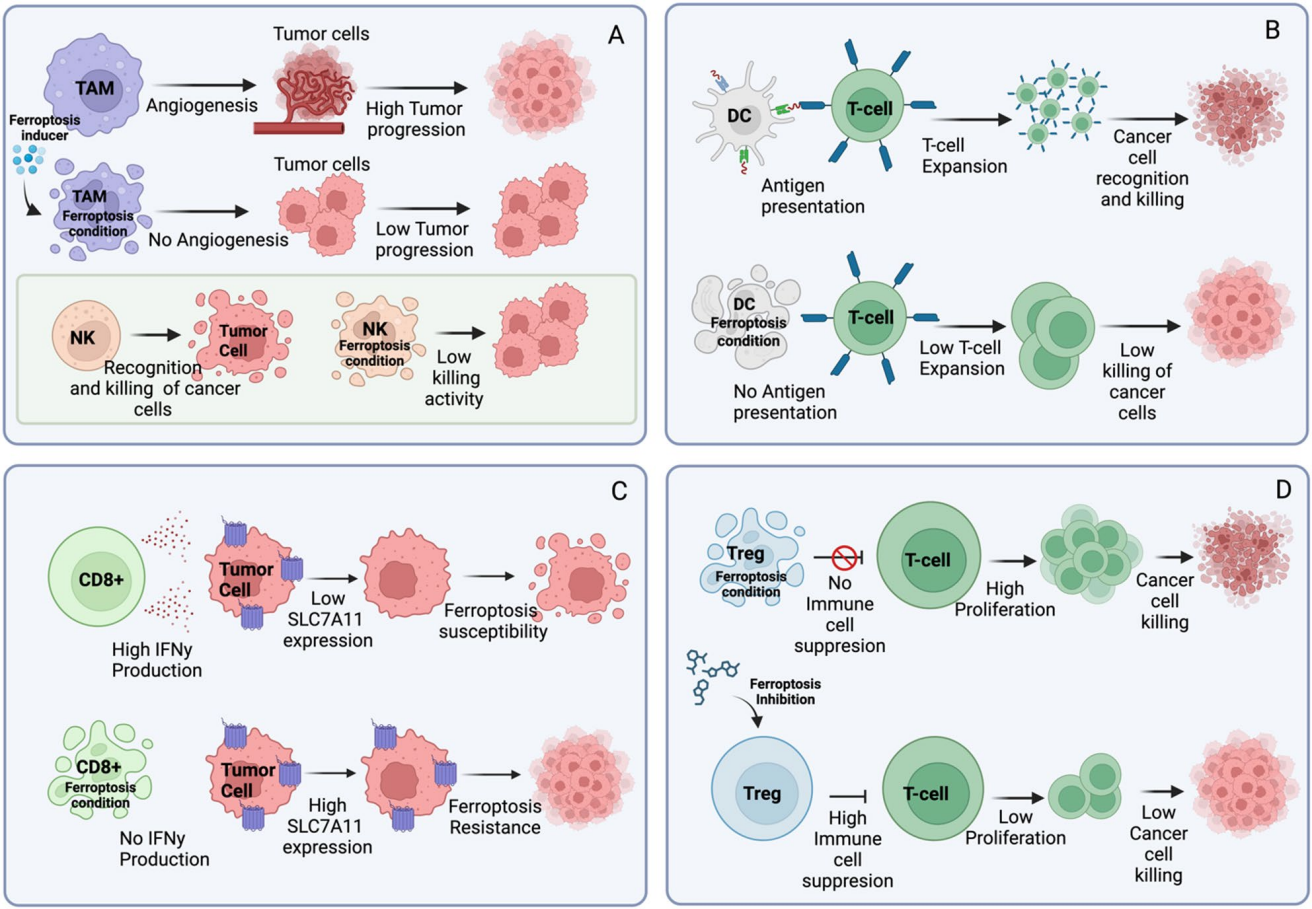
Ferroptosis in Immune cells: **A** TAM macrophages (Upper) can promote angiogenesis and metastasis of tumor cells. Induction of ferroptosis in TAM cells can reduce tumor progression (Middle). A similar situation can be described for NK cells (Down). **B** DCs are able to present the tumor cell antigen to T-cells. This mechanism favors the recognition of cancer cells. Under ferroptosis condition DC can’t present the antigen and the T-cells are unable to recognize cancer cells. **C** CD8^+^ T-cell present high IFNy production when activated. IFNy can reduce SLC7A11 expression in cancer cells leading them to higher ferroptotic sensitivity. When ferroptosis is induced in CD8^+^ T-cells, cancer cells maintain high SLC7A11 expression promoting resistence to ferroptosis and growth. **D** Treg cells subjected to ferroptosis mechanism limit their ability to suppress immune response, favoring T cell proliferation and the killing of cancer cell. Studies have shown that the inhibition of ferroptosis in Treg shows higher immune-suppressive power

Monocytes and Myeloid-derived suppressed cells (MDSC) are circulating cells that differentiate into macrophages and promote antitumor immunity by activating T-cells and tumor associated macrophages (TAM). At the same time these cells can support tumor progression through by promoting angiogenesis and metastasis. TAMs exhibit sensitivity to ferroptosis, with potentially a more immunosuppressive function when undergoing ferroptosis. The vulnerability of TAMs to ferroptosis allows them to be targeted and to enhance the efficacy of current cellular immunotherapies although the balance between anti- and pro-tumoral function needs to be considered [83] (Fig. 4A, upper panel). However, macrophages are highly plastic and, in response to stimuli in a specific environment, they will rapidly transform themselves into specific functional phenotypes, namely M1 type that exerts pro-inflammatory properties, and M2 type with anti-inflammatory and immunosuppressive properties [84]. This process is called polarization [85].

NK cells are involved in the elimination of tumor cells from primary tumor and distant sites. The explored role of ferroptosis in these cells shows a negative impact of ferroptosis induction, promoting a pro-tumorigenic function [86]. Different effects are exerted by ferroptosis on the cell compartment belonging to adaptive e immunity (Fig. 4A, lower panel).

Dendritic cells (DC)s exert a function of connection between the innate and adaptive immune system through antigen presentation to T-cells aimed to activation (48). Dysfunction of DCs results in tumor progression and is associated with accumulation of lipids. This effect can be caused by cancer cells dying from ferroptosis producing the inability to cross-present antigens showing a negative impact of ferroptosis [86, 87] (Fig. 4B).

T-cells anti-tumor activity exerted by CD8^+^ compartment involves the release of IFNγ which sensitizes cancer cells to ferroptosis, probably due to the ability of IFNγ to inhibit SCL7A11 [88]. At the same time, some studies have demonstrated that CD8^+^ cells are affected by ferroptosis more than cancer cells showing induction of lipid peroxidation. In contrast, CD4^+^ compartment is less vulnerable to ferroptosis and in particular, Treg subpopulation, shows a higher immune-suppressive potency when treated with ferroptosis inhibitors [89]. Thus, Inducing Treg death may be crucial to enhance anti-tumor immunity. A recent study found that inhibition of SLC7A11 induced ferroptosis and reverses the immunosuppressive function of [90, 91]. Furthermore, ferroptosis of Tregs induced by GPX4 inhibition, releases proinflammatory factors that promote DCs and CD8^+^ T-cells activation and enhance anti-tumor immune responses [89] (Fig. 4C, D).

Ferroptosis in B cells seems to be involved, but its role remains to be deeply investigated.

#### Ferroptosis-dependent TME modification

In the TME, immune cells are recruited and infiltrated in response to different factors [92] such as hypoxia, pH, nutritional deprivation [93, 94] and, under certain conditions, TME leads to the aggregation of immunosuppressive cells, such as MDSCs, Tregs and M2 TAMs [94]. Among the factors stimulating infiltration, hypoxia has been deeply studied [95, 96]. Hypoxia inducible factor-1 (HIF-1) is a heterodimeric protein stably expressed under hypoxia [97], and it drives the recruitment of immunosuppressive cells to the TME.

Furtheremore, tumor cells release several molecules under hypoxia such as chemokine ligands 2 and 5 (CCL2 and CCL5), CXC chemokines, prostaglandin E2 (PGE2) and TGF-β that can favor the recruitment of immunosuppressive cells. In this way, the TME leads to to impaired T-cell function. Recently, ferroptosis has been shown to have a relationship with several immune system cells in the TME, targeting these immunosuppressive cells. For example, Tregs can resist various environmental stresses, to exert immunosuppressive effects [98]. Another important role exerted by HIF-1 concerns the recruitment of macrophages, also driving them into M2 polarization, acquiring immunosuppression property [99, 100], as they have gained the ability to inhibit the activation of T-cells and DCs [101]. In this context, several studies have demonstrated that the induction of ferroptosis can revert M2 TAM in M1 polarization. Zero-valent iron nanoparticles (ZVI-NPs), for example, exerts the function of ferroptotic inducers and not only promote M1 polarization but may inhibit Tregs and promote anti-tumor immunity [85]. Other compounds involve MIL88B and RSL3 which, synergistically, induce TAM ferroptosis, activate several M1-related signaling pathways and decrease the M2 signaling expression [102]. Finally, the biomimetic molecule D@FMN-M, a new ferroptosis inducer, may induce M2 TAMs ferroptosis and promote M1 polarization through [103].

However, it is important to emphasize that the induction of ferroptosis in specific subtype of immune cells can only have beneficial effects in specific contexts. Indeed, although it can be a tool for enhancing the anti-tumor response, the use of ferroptotic inducers in other TME contexts results in inhibition of antitumor activity. In this regard, some studies have focused on compounds capable of inducing ferroptosis only in tumors cells [104]. Surprisingly, some molecules have been tested and found to be not only harmless versus immune cells, but, in some situation, can even support their proliferation. For example, Wang et al. demonstrated that Erastin, one of the most widely used ferroptosis inducers, promotes the proliferation and differentiation of Human Peripheral Blood Mononuclear Cells (PBMC). Specifically, they found that treating the cells with Erastin, through the metabolite released following lipid peroxidation, can inhibit bone morphogenetic protein (BMP) and activate the proliferation of B cell and NK cells [104].

The effectiveness of immunotherapy depends on the TME of tumors, which is related to the presence of specific metabolites and immune cells around tumor tissues. The TME can be characterized by two kinds of situations, called “cold tumors” and “hot tumors” [105]. In “hot tumors condition”, many anti-tumor T lymphocytes and other immune cells are infiltrated in TME, whereas “cold tumor” refer to tumors with few immune cells infiltration, forming a more compact and dense mass resistant to drugs penetration [106, 107]. Regarding the interaction with ferroptosis, “hot tumors” are not the best situation to sustain immunotherapy, since ferroptosis inducers often also act on immune cells, causing ferroptosis in immune cells, thereby reducing the effect of immunotherapy. Instead, in “cold tumors” the effect of ferroptosis-inducing agents combined with immunotherapy is highly effective and promising. However, other strategies focus on the possibility to promoting the infiltration of immune cells into the tumor mass, with the aim of transforming “cold tumors” into “hot tumors”, with the idea of allowing the immune system to reach even hidden cells where to induce ferroptosis [85].

Furthermore, it has been demonstrated that ferroptotic cancer cells release damage-associated molecular patterns (DAMPs) after death, and several studies have proven that they are able to activate immunological cell death (ICD), induced by recruited immune system cells [108]. For example, DAMPs can promote DC maturation and activation, activate adaptive immunity, and promote T cell initiation. DCs express pattern recognition receptors (PRRs) that mediate immune activation, recognizing specific signal such as DAMPs, and different PRRs react with specific DAMPs to activate specific pathways to prime T cells, leading to an adaptive immune response [109]. DC maturation upregulates costimulatory factors such as CD40, CD80, and CD86, all of which are required for T cell initiation. Several DAMPs released from dying cancer cells are emerging to have a pivotal role to support anti-tumor immunity. The most studied DAMPs involve high mobility group box 1 (HMGB1), calreticulin (CRT), adenosine triphosphate (ATP), and heat shock proteins (HSP) which are the most common DAMPs released by ferroptosis induction [110]. Once HMGB1 is released extracellularly, it promotes maturation of DC through the HMGB1/PI3K/Akt/mTOR axis and enhances the antigen-presenting ability of DCs by up-regulating markers on DCs [111, 112]. ATP, an essential nucleotide for metabolism, is released after cancer cells ferroptosis in the early stage and is recognized by DC through P2RX7 receptor. In turn, DCs secretes IL-1β and induce CD8^+^ T-cell polarization. Heat shock proteins HSPs are exposed to the surface of cancer cells during ferroptosis, and DCs cells interact with HSP to promote their maturation and the production of several cytokines to stimulate anti-tumor immunity [113].

Lipid peroxidation also plays a crucial role in inducing immune cell activity. Indeed, oxidation produces L-OOH markers of “modified-self” type and the generation of these altered lipids, as well as oxidized lipid-proteins, creates the so-called “neo-self determinants” that are recognized by specific innate and adaptive immune responses through various receptors of innate immunity, including scavenger receptors expressed on macrophages, tall-like receptors expressed on DCs [114] and natural antibodies. In general, these lipidic neo-antigens are recognized in specific manner, in which epitopes are formed on different endogenous proteins and lipids (e.g., PLs like PE containing a free amino group). For example, when phosphatidylcholine (PC) present in lipid membrane and cell membranes is oxidized, decomposition of the unsaturated fatty acid generates a spectrum of reactive molecules, such as malondialdehyde (MDA) and 4-HNE [115]. All these products are highly reactive and can form neo-epitopes.

## Main text

Revealing ferroptosis mechanism of action in cancer cells has opened up new possibilities in cancer treatment and has recently enabled the use of a novel therapeutic approach involving the administration of ferroptosis inducer with immunotherapy. Here we show the actual combined therapy between ferroptosis inducers and immunotherapy compounds for the treatment of several tumor types.

### Ferroptosis induction and immune check-point inhibitors cooperation

T lymphocytes, the most studied immune cells, represent the most important effector cells, especially in the CD8^+^ population, that has a close interaction with tumor cells. T-cells activity can be regulated by co-stimulatory or co-inhibitory signaling pathways, just like the co-stimulatory factors of activated T-cell surface that are known as the immune check-points [110]. Several kinds of immunotherapies have been used for the treatment of cancer, including cellular and gene immunotherapy, which directly uses immune cell effectors, and immunotherapy based on immune check-point inhibitors (ICI)s. The latter, generally focused on targeting, with the use of specific drugs, ligand-receptor interaction, has revolutionized the clinical treatment of various cancer patients. ICIs act by activating potent cytotoxic T-cells to drive anti-tumor immunity [116]. Some ICIs have been approved as cancer immunotherapy, as they target CTLA4/CD80, or PD-1/PD-L1 interaction [117]. 

### Different strategies to enhance anti-PD-1-antibody immunotherapy inducing ferroptosis

In recent years, ferroptotic mechanisms have been widely studied, and, aside from the common ferroptosis markers, many non-canonical-ferroptosis factors have been discovered with the ability to repress or potentiate ferroptosis in a more or less indirect way. Various strategies can involve both canonical and non-canonical factors, in order to test them as target to evaluate their capacity to modulate the ferroptotic mechanism and their contribution to improving the immunotherapy response based on anti-PD-1-antibody in particular (Table 1).

**Table 1:** Current ferroptosis inducer and immune check-point inhibitors combined therapies

Co-therapy with PD-1	Effects	Cancer Types	References
Inhibition of GPX4	Elevation of ferroptosis biomarkers, increases of percentage of activated immune cells	Triple negative breast cancer (LAR subtype)	[118]
Inhibition of FSP1 combined with photodynamic therapy	Induction of immunogenic cell death, release of tumor antigen to promote dendritic cell (DC) maturation and cytotoxic T lymphocyte stimulation	Colon cancer	[119]
Creatine supplementation	Enhancement of CD8 + T cell infiltration, promotion of ferroptosis	Colorectal cancer	[120]
Inhibition of FSP1 combined with sonodynamic therapy by using nanoparticles	Promotes lipid peroxidation, triggering immunological cell death	Lung cancer	[121]
Inhibition of DHODH	Increased of CD8 + T cell infiltration and Increased of IFNg production, CD69, CD107a and Granzyme B expression. Increase of Lipid peroxidation	Melanoma	[122]
Inhibition of PGAM1	Promotes CD8 + T-Cell Infiltration and downregulates PD-L1	Hepatocellular carcinoma	[125]
Depletion of Membrane cholesterol by Engineered Nanozyme	High level of lipid peroxidation, high percentage of TAM M1, high CD8 + cytotoxic activity	Breast Cancer	[127]
TXNRD targeting by miR-21-3p-Loaded Gold-Nanoparticles	High lipid peroxidation, decreases tumor progression	Melanoma	[133]
Inhibition of BRD4	Enhances ferroptosis, increases of CD8 + T cells INFγ and Granzyme B	Melanoma	[136]
Introduction of m6A methylation on the mRNA of ferroptotic markers and immune checkpoint molecules	Increases ferroptosis, improves immunological response	Strategy under investigation	[139–142]
Inhibition of TRIM34	Increases ferroptosis, sensitizes tumor cells to anti-PD1 immunotherapy	Hepatocellular carcinoma	[143]
Inhibition of USP8 in combination with SAS	High CD8 + T cell infiltration	Colon tumor	[149]
MEF	Upregulation of LPCAT3 and ACSL4. Enhancement of downstream factors of the IFNγ signal	Melanoma	[151]
Inhibition of CPT1A	Improvement of immune cell response, infiltration of T Cells	Lung Cancer	[154]

### Canonical ferroptosis pathways

#### GPX4-dependent ferroptosis protection

Regarding “canonical ferroptotic factors”, the most used strategy involves the inhibition of GPX4, the most important protecting factor against ferroptosis. In triple negative breast cancer (TNBC) the inhibition of GPX4 was tested to enhance immunotherapy. TNBC is a biologically and clinically heterogeneous disease classified into several subtypes. Among them, the luminal androgen receptor (LAR) subtype of TNBC is characterized by the upregulation of enzymes involved in GSH metabolism, particularly GPX4. Several data indicated that GPX4 is a potential factor of GSH metabolism that suppresses ferroptosis in LAR tumors. In fact, androgen receptor (AR) inhibitors strongly reduce GPX4 expression, demonstrating that AR promotes and drives GPX4 expression in LAR subtype of TNBC tumor. For these reasons, the researchers tested the anticancer effect of GPX4 inhibitors in AR-expressing TNBC tumors in vivo. The effect of GPX4 knockdown or utilization of GPX4 inhibitors effectively led to the elevation of ferroptosis biomarkers and the recruitment of different types of effector immune cells, among which CD4^+^, CD8^+^, and CD86^+^ cells and the proportion of M2 macrophages. Therefore, in this study, ferroptotic induction is evaluated in combination with immunotherapy using GPX4 inhibitors in LAR tumors. In an in vivo model, using tumors xenograft with breast cancer cell lines in mouse, GPX4 inhibitors plus anti-PD-1-antibody significantly inhibited tumor growth compared with therapy with anti-PD-1 therapy alone. Despite the number of infiltrated immune cells is like the control conditions, the percentage of activated immune cells significantly increased in combined therapy compared to the monotherapies. SLC7A11 levels also decreased with combined treatment, suggesting that the combined therapy acts in different ways that may involve other branches of the ferroptosis pathways. In support of the possibility of this therapy in humans, analysis of a cohort of 21 patients with TNBC undergoing neoadjuvant immunotherapy revealed that TNBCs with higher AR expression showed enhanced iron metabolism, unsaturated FA metabolism, and GSH metabolism activity. However, it’s important to highlight that these in vivo preclinical experiments showed safety, opening the door to clinical testing as a strategy for LAR tumors subtypes [118].

#### GPX4-independent ferroptosis protection

Engineered photosensitive LNPs could trigger synergistic ferroptosis through FSP1 gene silencing and ROS generation in colon cancer cells, inducing immunogenic cell death and tumor antigen release to promote DCs maturation and cytotoxic T lymphocyte stimulation. Indeed, after photosensitive LNPs treatment, cell membrane disruption is promoted, thus causing DAMPs release to stimulate subsequent immune activities. Specifically, they found higher level of HMGB1 as well as calreticulin (CRT), molecules known to induce immunogenic response. This study demonstrates a synergistic potential between FSP1 gene silencing, photodynamic therapy and immune cells activity expanding the applications of photosensitive LNPs in clinical cancer treatment [119].

Another study performed by Zhou et al. demonstrates that Creatine is involved in FSP1-dependent-ferroptosis mechanism and potently induces ferroptosis in CRC. Briefly, creatine binds extracellular signal-regulated kinase 2 (ERK2), reducing its activation by mitogen-activated protein kinase kinase 1 (MEK1). By inhibiting the transport of creatine, which targets SLC6A8 transporter, ERK2 is activated, which in turn, phosphorylates FSP1 and stabilizes it to inhibit ferroptosis. Creatine supplementation suppresses tumor growth, enhances CD8 + T cell infiltration, and sensitizes tumors to PD-1 immunotherapy. In a murine model of CRC, they found that creatine administration promoted tumor cell ferroptosis and enhanced the number and activation of tumor-infiltrating T cells, synergistically enhancing the efficacy of immune check-point blockade (ICB) therapy. In summary, their work identifies the SLC6A8-creatine-ERK2-FSP1 axis as a previously unknown immune-evasion mechanism for CRC in the TME, confirming that targeting anti-ferroptotic pathways is a promising therapeutic option as it induces immune cells activity and improves ICB therapy in preclinical experiments [120].

A different strategy has proposed the use of sonodynamic therapy (SDT) to induce lipid peroxidation, ferroptosis and thus enhance immunogenic cell death. Chen et al. used nanoparticles (NPs) which combinate cell-membrane targeting sonosensitizer able to generate ROS under ultrasound treatment (TBT-CQ1) with FSP1 inhibitor, facilitating cell-membrane targeting sonodynamic-triggered ferroptosis. In this way NPs could induce a sonodynamic effect, which promotes lipid peroxidation, suppresses FSP1, induces CoQ10 depletion, and finally induce ferroptosis. In vitro results demonstrated that synergetic cell membrane targeting SDT/FSP1 inhibition triggered immunogenic cell death (ICD). Moreover, this treatment inhibited the tumor growth and simultaneously activated antitumor immunity to suppress lung metastasis. This work represents a novel alternative therapy combining cell membrane targeting SDT and FSP1 inhibition, potentially inspiring future research in the development of effective cancer therapies based on SDT/ferroptosis synergy [121].

Recent experiments reported that inhibition of DHODH expression in mouse cancer cells inhibited the growth of tumors in immune competent mice, but not in immune-deficient NSG mice. The authors observed higher levels of CD8^+^ T cell infiltration and a higher percentage of IFNγ^+^ CD8^+^ T cells compared to DHODH WT control tumors. The administration of BRQ, an inhibitor of DHODH, showed the same results. These results strongly suggest that the stimulation of antitumor immunity is critical for the anti-tumor effects of DHODH inhibition. Pretreating the tumor cells with BRQ increased levels of CD69, IFNγ, CD107a, and granzyme B expression in co-cultured CD8^+^ T cells, consistent with higher levels of cytotoxicity. Then, they discovered involvement in ferroptosis mechanism upon DHODH inhibition by measuring lipid peroxidation of tumor cells in the presence or absence of CD8^+^ T cells. Exposure of tumor cells to activated T-cells led to increase of lipid peroxidation levels, but they were further increased by pretreatment of tumor cells with BRQ in the presence of CD8^+^ T cells. Importantly, treatment of tumor cells with the inhibitor of ferroptosis Fer-1, significantly reversed the increased sensitivity to CD8^+^ T cell-mediated killing in both BRQ treated and DHODH knocked-out (KO) cells. Through lipidomic and metabolomic analyses, they revealed a link between DHODH and CDP-choline level. DHODH inhibition reduced CDP-choline levels, and its subsequent impact on the synthesis of various phosphatidilcholine (PC) species contributed to the effect of DHODH inhibition on ferroptosis induction. In fact, CDP-choline supplementation was able to reverse the effects of both DHODH inhibitors and DHODH KO on inducing lipid peroxidation. Finally, they tested whether BRQ may enhance the efficacy of anti-PD-1 checkpoint blockade in murine tumor. Although treatment with low dose of BRQ alone did not significantly affect tumor growth, the combination between BRQ at the same dose and anti-PD-1 antibody strongly reduced tumor growth compared to either treatment alone [122].

### Non canonical ferroptosis pathways

#### PGAM1 Inhibition

An example of the effectiveness of the cooperation between anti-PD-1-antibody and a “non-canonical ferroptotic- actors” regards the targeting of phosphoglycerate mutase 1 (PGAM1), an enzyme overexpressed in various human cancers, including hepatocellular carcinoma (HCC) [123] and which plays a crucial role in tumor progression through its metabolic mechanism [124]. Its pivotal role is to catalyze the conversion of 3-phosphoglycerate (3-PG) to 2-phosphoglycerate (2-PG). In addition, other carcinogenic effects of its activity unrelated to its canonical role have been demonstrated. In fact, high PGAM1 expression correlates with a poor prognosis in HCC patients and attenuates the infiltration and activation of CD8^+^ T-cells. A recent study showed that PGAM1 controls ferroptosis, in fact its inhibition increases ROS level and decreases GPX4 expression. Interesting, PGAM1 Inhibition could moreover promote CD8^+^ T-Cell Infiltration and downregulate PD-L1 in HCC. Experiments performed in PDX mouse model have tested EH3, a selective PMAG1 inhibitor, with PD-1 blockade immunotherapy. They observed a strong co-therapy effect. Analysis of HCC patients treated with immunotherapy, who demonstrated an enlargement of tumor size, had higher PGAM1 expression as well as lower CD8 expression. These results suggested that HCC patients with low PGAM1 expression might benefit more from anti-PD-1 immunotherapy. Therefore, patients with high expression of PGAM1 can be benefitted by the development of strategic co-therapy [125].

#### Cholesterol depletion

Another strategy investigated by the researchers involves targeting membrane cholesterol. It has been demonstrated that high level of cholesterol impedes the diffusion of lipid peroxidation, inhibiting ferroptosis [126]. Furthermore, cholesterol can upregulate the expression of PD-L1 in cancer cells [127] and, on the other hand, induce CD8^+^ T cells exhaustion. Cholesterol depletion using cholesterol oxidase (ChOX) is a strategy proposed by Bai T et al. to enhance ferroptosis-immune therapy. The strategy involves the use of engineered nanoparticles with biological enzyme-mimicking properties, referred to “nanozymes” [128, 129]. They named their nanozyme “Fe-MOF/CP”, since it is composed of iron metal-organic framework (Fe-MOF) nanoparticles, biological ChOx, and PEGylation, for integrated ferroptosis-immune tumor therapy in breast cancer cell line. They demonstrated that Fe-MOF/CP induced high level of cellular lipid peroxidation, 4-HNE and low levels of GPX4 improving ferroptosis in cancer cells. Moreover, Fe-MOF/CP-induced antitumor immune responses have been systematically studied. Recent data focused on the role of TAM (M1 and M2) in antitumor therapy, and it was demonstrated that cholesterol promotes tumor progression by converting M1, which has high cytotoxic activity, to the M2 phenotype, without killing activity. Accordingly, Fe-MOF/CP treatment increased the M1 portion of macrophages. Observations from similar experiments showed that the treatment with nanozymes also enhanced the percentage of mature antigen-presenting DCs in the mouse model and the cytotoxic activity of CD8^+^ T cells. Starting from these findings, the combined therapy between Fe-MOF/CP and anti-PD-1-antibody has been tested. In the mouse model, Fe-MOF/CP + anti-PD-1 antibody significantly inhibited both primary and distant tumors and showed higher survival rate. This work underlines the potential of Fe-MOF/CP as a multifunctional therapeutic agent that integrates ferroptosis and immunotherapy for cancer treatment. With its intrinsic action to deplete cholesterol, this nanozyme impairs the resistance mechanisms of cancer cells, allowing the way for more effective and durable cancer therapies. This research is intriguing and provides a safe and efficient strategy for the development of advanced treatments that synergize ferroptosis and immune responses to fight cancer [127]. The use of nanoparticles has become widespread in recent years, and many studies are dedicated to the development of this transport methodology.

#### miRNAs delivery

Studies demonstrated that miRNAs are involved in ferroptosis mechanisms [130, 131]. Some examples sustain these discoveries: miR-137 exerts its inhibitory effect on ferroptosis through the suppression of glutamine transporter SLC1A5 in melanoma cells [132]. SLC1A5 is recently related to ferroptosis since glutamine is a precursor of GSH [132]. In addition, it has been observed in gastric cancer that miR-522 secreted by fibroblasts suppresses ferroptosis of tumor cells, significantly contributing to chemo-resistance. In 2022 Guo et al. [133] suggested a potential co-therapy between immunotherapy and a microRNA. They obtained a miRNAs expression profile in IFNγ-driven ferroptosis by RNA sequencing in human melanoma cell lines. Among them, they focused on miR-21-3p. Through in vivo experiments in mouse model with B16F10 melanoma cells, they observed increasing level of mir21-3p after anti-PD-1 antibody treatment. The scientists focused on TXNRD, an enzyme with a key role in the regulation of redox hemostasis [134]. The study demonstrated that TXNRD1 was a novel direct target of miR-21–3p in human melanoma cells. They showed that the activity of miR-21-3p on TXNRD1 was able to generate ferroptosis in melanoma by enhancing lipid peroxidation. Starting from these data they evaluated the combined effect of tumors miR-21-3p overexpression and anti-PD-1 observing a clear decrease in tumor progression. This effect was reversed by ferroptotic inhibitor, showing that the effect of the therapy was ferroptosis-dependent. Since they found excellent in vitro results, they tested their translational potential, using nanoparticles as delivery system for melanoma cells. In their experiments, gold nanoparticles loaded with miR-21-3p act synergistically with anti-PD-1 immunotherapy in melanoma cells in vitro. These data indicated that nanoparticle-based transfection as a vehicle for miR-21-3p increases miR-21-3P intracellular expression and sensitizes to anti-PD-1 immunotherapy by facilitating ferroptosis [133].

#### Epigenetic modifications

##### BET inhibitors

The idea of ​​involving and targeting the epigenetic system as a ferroptotic regulator is intriguing. A recent study investigated the efficacy of the bromodomain inhibition, a domain of specific proteins able to recognize acetylated lysine residues such as those on the tails of histones, and bromo-domain and extra terminal (BET) inhibitors, to target ferroptosis at chromatin level. Indeed, through a specific algorithm, they found that BET inhibitors were associated to ferroptosis activity. Moreover, colony-count experiments revealed a strong synergy in melanoma cells when BET inhibitors were combined with the ferroptosis inducer RSL3. Specifically, they noted that the overexpression of BRD4, a member of BET family, upregulated a metabolic enzyme, called Aldo-Keto Reductase family 1 member C2 (AKR1C2), in melanoma cells. The same authors demonstrated in their previous study the involvement of AKR1C2 on ferroptosis inhibition by degrading lipid peroxides [135]. Consistently, inhibition of AKR1C2 sensitized melanoma cells to ferroptosis and the genetic inhibition of BRD4 enhanced the ferroptosis induced by RSL3 suggesting that BET inhibitors sensitize melanoma cells to ferroptosis. Furthermore, they found a potential correlation with anti-PD-1 antibody as efficient co-therapy. BET inhibitor treatment potentiated the efficacy of anti-PD-1 antibody, increasing the number of CD8^+^ T cells producing INFγ and Granzyme B in vivo. They conclude that blocking BRD4 via BET inhibitors sensitizes melanoma to GPX4 inhibition-induced ferroptosis and immunotherapy in vivo [136].

##### m6A modification

Multiple investigations have established a connection between the introduction of m6A modification on mRNA and programmed cell death, specifically ferroptosis [137, 138]. m6A modification determines prevalent post-transcriptional modification in human mRNA, having critical functions in regulating mRNA stability. Since these modifications can be added, erased and read by different factors, m6A methylation can manifest an increase or decrease in mRNA stability depending on the context. Several ferroptotic factors can be targeted by proteins that add m6A group on mRNA. A recent study has discovered that the expression of the two components of xCT system, SLC3A2 and SLC7A11, can be modulated by proteins involved in m6A modification, YTHDC2 and METTL3, leading to the induction of ferroptosis in lung adenocarcinoma cells [139, 140]. METTL3 can also target GPX4 by introducing m6A modification on its mRNA, increasing its mRNA stability in breast cancer and showing a pivotal role in anti-ferroptotic mechanism in that context. Another target of m6A modification is represented by Nuclear factor erythroid 2-Related Factor 2 (NRF2), a well-known ferroptotic effector, methylated by WTAP on the 3’UTR region. NFR2, being a transcription factor, can directly interact with SLCA11 promoter and activate its transcription. Numerous evidence has demonstrated that the mechanisms concerning the methylation of m6A are controlled in several kinds of tumors and are also significantly associated with the modulation of the expression of checkpoint such as PD-1 and its ligand. For examples, two other proteins involved in m6A methylation, FTO and IGF2BP, exhibit key roles on the stability of the mRNA of PD-1. On the other hand, METTL3 controls PD-L1 expression on breast cancer favoring the antitumor activity of the immune cells [141]. Recent studies correlated the lack of METTL3 with the improvement of the immunological responses mediated by the treatment with anti-PD-1-antibody [142]. The discovery that SLC7A11 can be targeted by m6A modification, and that CD8^+^ T-cells can modifiy SLC7A11 expression, and the other evidence regarding the role of m6A methylation in ferroptosis and immune cells activity, suggested that m6A mediated ferroptosis can be used as a co-therapy to improve immune cells activity. red. However, it is important to note that the co-therapy strategies based on m6A-induced ferroptosis have not yet been extensively tested. Thus, based on the involvement of m6A modification in ferroptosis mechanisms, the idea of a co-therapy as a novel strategy for cancer treatment to promote m6A mediated ferroptosis to sensitize cells and potentiate the efficacy of immunotherapy is attractive, and scientists believe that research should focus on targeting m6A, innovating immunotherapy and implementing it in the near future [142].

##### Ubiquitination

Yao f. et al. [143] described a ubiquitin-dependent mechanism to enhance immunotherapy efficacy via ferroptosis. Using CRISPR/Cas9 screening they identified a ubiquitin ligase that might suppress ferroptosis and focused on TRIM34, as the most negatively related gene. Primary in vitro experiments demonstrated that TRIM34 suppressed ferroptosis in HCC cells, by reducing ROS and intracellular level of iron. They showed that TRIM34-anti-ferroptotic activity depends on its downstream target, UPF1, a core component of the nonsense-mediated mRNA decay (NMD) pathway, which has been already associated with ferroptosis. Through this study, by finding that UPF1 targets GPX4, the authors demonstrated that TRIM34 impedes ferroptosis in HCC by degrading UPF1 to enhance GPX4 mRNA stability. The results were confirmed in vivo, with orthotopic transplantation of HCC model in C57BL/6 mice. Furthermore, in vivo experiments showed that TRIM34 promotes T-cells infiltration and increased the number of activated CD8^+^ T-cells, prompting the authors to test the enhancement of TRIM34-dependent ferroptosis in cooperation with immunotherapy. Silencing of TRIM34 in tumors injected in mouse model with anti PD-1 increased ferroptotic markers and sensitized tumor cells to anti-PD-1 immunotherapy [143].

The role of DUBs has been deeply studied in cancer, and their inhibitors also seem to provide high response for cancer treatment in vivo [144]. Ubiquitin specific protease 8 (USP8), which has a pivotal role in cell division and tumorigenesis [145, 146] shows the ability to remove the ubiquitination from a target protein, protecting it from the degradation [147]. USP8 has also been identified as being involved in the mechanisms of resistance to ferroptosis in liver cancer. Furthermore, a new study linked USP8 to ferroptosis mechanisms also in colorectal cancer context. Indeed, one of the targets of USP8 is GPX4, controlling the fate of this important ferroptosis protector. The data showed that GPX4 reduces its protein but not mRNA expression upon USP8 inhibition. One of the drugs approved by the food and drug administration (FDA) for the use in clinical treatment is “sulfasalazine” (SAS). SAS has also recently been identified as a potent ferroptosis inducer [148], and it is therefore used in this study to induce ferroptosis as a combined therapeutic treatment. When the scientists inhibited USP8 in combination with SAS, they revealed a reduction in tumor growth and high CD8^+^ T-cells infiltration. For this reason, they tried a combined treatment that involves also PD-1 blockade in vivo. So, they performed a triple combined therapy involving USP8 inhibitor (DUB-IN-2), SAS and anti-PD-1-antibody observing an increased in tumor infiltrating CD8^+^ T-cells and a reduction of murine colon tumor, selected as in vivo model, in a strong significant statistically manner, compared to the other conditions. This study suggests that it is possible to target ferroptosis in different ways simultaneously, and this leads to an amplifying response of the anti-PD-1 antibody [149].

### IFNy-dependent ferroptosis induction

Finding compounds that can induce ferroptosis whose safety has already been approved by the FDA may be a strong point for their use as a therapy. Among the compounds already approved by FDA, some of them have been tested as inducers of ferroptosis. Mefloquine (MEF) is a quinoline ethanol used for the treatment of Malaria [150]. It has been found as a novel inducer of ferroptosis, because studies associated its activity with an increase of lipid peroxidation in melanoma and lung cancer. However, MEF has already been associated to anticancer activity and its ability to affect immune response is unclear. A study demonstrated that the administration of MEF with INFγ upregulates a key molecule for fosfolipidic production and lipid peroxidation, lysophosphatidylcholine acyltransferase 3 (LPCAT3) and, with minor effect, on ACSL4. They observed that MEF potentiates the factors downstream of the IFNγ signal: p-STAT1 and IRF1. Through co-culture experiments between CD8^+^ T cells, melanoma and lung cancer cells they found a combined effect between MEF and anti-PD-1 antibody. The killing activity of CD8^+^ T-cells was significantly increased in the combined treatment comparing with MEF or anti-PD-1 alone. The results were confirmed in vivo, in mouse model, where a gain of T-cell function in combined condition was observed. They concluded that MEF, by enhancing ferroptosis, improves the therapeutic efficacy of anti-PD-1 through INFγ-IFR1-LPCAT3 axis, suggesting MEF as a promising sensitizer for anti PD-1 immunotherapy. In accordance with this, they observed a high expression of LPCAT3 in tissues of melanoma patients treated with anti-PD-1 [151].

#### Metabolic contribution

Recent findings have also shed light on the metabolism contribution to resistance of ferroptosis [152, 153]. In some situations, manipulation of metabolic pathways has been associated with ferroptosis sensitivity, and which is easily understandable since ferroptosis depends on fatty acid production and the availability of specific amino acids. Among the complex pathways related to fatty acid oxidation (FAO), the enzyme Carnitine Palmitoyl Transferase 1 A (CPT1A) plays a major role in this metabolic pathway and controls the transport of long-chain fatty acid into the mitochondria, the site of β-oxidation of fatty acids. In an interesting recent study, bioinformatic analysis revealed that CPT1A can also control ferroptosis markers, including NRF2, GPX4 and ACSL4. Further investigations have found a strong association with the c-Myc oncogene in lung cancer, demonstrating that CPT1A indirectly regulates c-Myc expression. In fact, CPT1A has been found to interact with c-Myc, acting as a competitor for the binding of a ubiquitin ligase, compromising this interaction and thus improving the stability of c-Myc mRNA. Since c-Myc has been found to regulate NRF2/GPX4 cellular system, the CPT1A indirectly gives a contribution to suppressing ferroptosis in lung cancer. For these reasons, combined therapy including the inhibition of CPT1A has been tested to enhance immunotherapy. In the mouse model, the combined treatment of CPT1A inhibitor and anti-PD-1 antibody resulted highly effective in inhibiting tumor growth. The therapy reduces ferroptosis by acting on the increase of GPX4 activity, reduces the levels of PUFAs produced by ACSL4 and improves immune cells response. Notably, the therapy targets both T-cells infiltration and/or activation as well as increasing the percentage of TAM M2, as revealed by experiments on murine models [154].

According to the data explained here, it results evident that acting on the mechanism of ferroptosis clearly improves existing immunotherapy with regard to ICIs, in particular anti-PD-1 antibody. This is true across various tumor types and the literature shows that targeting different mechanisms of ferroptosis using different strategy leads in any case to the success of co-therapy with anti-PD-1. Although these data are currently limited in preclinical experiments, this evidence encourages researchers to improve these aspects and open the door to the translational applicability of co-therapy as a promising strategy to overcome tumor cell resistance to the immunotherapy in many tumor contexts.

### Ferroptosis contribution to ATMPs activity

#### CAR T-cells

In the Advanced Therapy Medicinal Products (ATMPs) scenario, CAR T-cells, which are patient-specific T-cells engineered to express receptors that specifically target cancer antigens, have changed the therapeutic landscape of hematological malignancies with overwhelming success. Nevertheless, albeit some important results were obtained also in neuroblastoma [155, 156], CAR T-cells have demonstrated poor or limited efficacy in solid tumors. The immunosuppressive nature of the TME and the poor expression of tumor-specific antigens, selectively and uniformly produced by tumor cells, constitute the greatest impediment to CAR T-cells treatment. To overcome these significant challenges, new strategies need to be improved to enhanc the efficacy of CAR T-cells in clinical applications for blood and solid malignancies. Combining CAR T-cells therapy with ferroptosis inducers shows a promising anti-tumor activity, particularly in solid tumors. This approach leverages ferroptosis to increase the efficacy of CAR T-cells treatment by promoting tumor cell death and enhancing immunogenicity. In particular, the ferroptosis-inducing agents can create a pro-inflammatory environment that can further enhance the efficacy and ability of CAR T-cells to recognize and destroy cancer cells, thus leading to better therapeutic outcomes.

Gao Y. et al. demonstrated that IFNκ can enhance the sensitivity of tumor cells to ferroptosis, and that CAR T-cells engineered to secrete IFNκ showed increased antitumor efficacy in vitro and in vivo [157]. Based on previous studies, which already reported that the IFN family members could induce ferroptosis [158, 159], they demonstrated, using human lung cell lines, that IFNκ can sensitized tumor cells to ferroptosis. Furthermore, they showed, in vitro, how IFNκ increases the cytotoxic activity of CAR T-cells. The authors explored the effect of CAR T-cells on antigen-negative tumors engineering the IFNκ into a CD276-CAR vector for establishing IFNκ-CD276-CAR T-cells. The results obtained showed how the IFNκ increased cytotoxicity in both H460 cells (antigen positive) and H322 cells (antigen-negative). Moreover, they observed that the release of perforin and Granzyme B and CD69 expression were upregulated in the IFNκ-CD276-CAR T-cell group. In contrast, the authors observed no differences in cell differentiation and proliferation in vitro between the two groups analyzed (IFNκ-CD276-CAR T-cell and CD276-CAR T-cell). In several mouse models, they observed enhanced CAR T-cell persistence in vivo and antitumor efficiency in an IFNκ dose-dependent manner. Furthermore, they evaluated whether the effects of IFNκ could be extended to other CAR T-cells. Interestingly they found that IFNκ-NKG2D-CAR T-cells showed positive results as well as IFNκ-CD276-CAR T-cells. In this paper, the authors showed that IFNκ induces ferroptosis and enhances the CAR T-cells antitumor effects.

Receptor tyrosine kinase-like orphan receptor 1 (ROR1) is a member of the type I receptor tyrosine kinase (RTK) family [160] that shows low or minimal expression in healthy (adult) tissues whereas is highly expressed in various tumor cell types [161, 162]. ROR1 is expressed in the 42% of patients affected by lung adenocarcinoma with 38% showing high levels of expression [163]. It has been observed that, in several cancers, ROR1 expression in tumor cells is linked to a dismal prognosis [164]. Altogether these data prompt ROR1 as a potential target for the treatment of non-small cell lung cancer (NSCLC). In a recent paper, Li et al. demonstrated that ferroptosis inducers combined with ROR1 CAR T-cells enhanced anti-tumor efficacy in NSCLC by promoting ferroptosis through increased lipid peroxidation [163]. By treating NCI-H1299 cell line and primary tumor cells (obtained from patients) with the ferroptosis inducer RSL3, the authors observed increased sensitivity to IFNγ secreted by ROR1 CAR T-cells. Moreover, they showed that ROR1 CAR T-cells enhanced the production of phosphatidylcholine with diacyl-polyunsaturated fatty acid tails (PC-PUFA2) working in tandem with IFNγ. Interestingly, this enhancement promoted the expression of ACSL4, which in turn boosted the overall antitumor response. To evaluate the therapeutic potential of the combined treatment in vivo, the authors developed a human metastatic NSCLC mouse model. They found that the combination of ROR1 CAR T-cells and RSL3 exhibited an important therapeutic efficacy in attenuating metastatic progression. Furthermore, they found that the combined approach led to a significant extended survival of treated mice compared to those receiving RSL3 or ROR1 CAR T-cells treatment alone. Taken together the results reported in this paper show how the combined treatment of the CAR T-cells with ferroptosis inducers can enhance anti-tumor efficacy in NSCLC and significantly improve survival outcomes in this NSCLC metastatic model.

In a recent publication, Manara P. and collegues [164] found that translation disruption, by inhibition of NRF2 translation via cap-dependent initiation blockade, sensitizes diffuse large B-cell lymphoma (DLBCL) to ferroptosis, enhancing the efficacy of CAR T-cells. They found that Zotatifin, an eIF4A inhibitor, showed pronounced synergy with several ferroptosis-inducing compounds across multiple cellular systems both in vitro, DLBCL cell lines, and in vivo, in DLBCL patient-derived xenograft tumors.

However, when the compounds were used in combination, an important host toxiticy (in vivo) was observed. Hence, they performed a dose optimization, in terms of frequency of treatments, to manage the toxicity. Furthermore, they performed an experiment of dose escalation of Zotatifin, evaluating the viability in three different exposure times, in CAR T-cells combined treatment. Unfortunately, they found that Zotatifin significantly reduced viability, including complete death at higher concentrations and exposure times, of CAR T-cells when used as a combined treatment. This finding suggests that the co-treatment Zotatifin/CAR T-cells is therapeutically incompatible. However, by exploring different co-treatment conditions, they found that cells treated for 24 h before adding CAR T-cells (they performed a washout after the treatment) were significantly more responsive to CAR T-cells than the control (DMSO-pretreated cells).

It is important to note that the drug’s in vivo half-lives made washouts and timing more challenging, so the authors observed only transient sensitization, nevertless the co-treated animals achieved a prolonged survival. In this report, as also showed by Li et al. [163], they found a strong synergy between Zotatifin and IFNγ in lymphoma cell lines, suggesting that Zotatifin can sensitize tumors to CAR T-cell-mediated ferroptosis in a manner similar to pharmacologic inducers. This study highlighted the Zotatifin’s ability to prime tumor DLBCL cells for ferroptotic stress, driving them to enhanced susceptibility to CAR T-cells cytotoxicity.

The promising results obtained from combining CAR T-cells with ferroptosis inducers (either as co-treatment or as sequential treatment, with a ferroptosis inducers agent followed by CAR T-cells exposure) suggest that this approach may enhance cancer treatment by improving CAR T-cell function and overcoming immunotherapy resistance. Interestingly, this approach can potentially expand the therapeutic opportunities for CAR T-cells therapy in solid tumors. Notewhorty each study has showed a different and specific mechanism of action in the antitumor activity obtained by the combined treatments. Gao et al. showed, in lung cancer models, how IFNκ, in combination with arachidonic acid, can sensitize tumor cells to ferroptosis via the IFNAR/STAT1/ACSL4 axis, and the cytotoxic activity of CAR T-cells. Li et al. showed how the combination of ROR1 CAR-T cells with ferroptosis inducers enhanced anti-tumor efficacy in NSCLC by promoting ferroptosis through increased lipid peroxidation. Finally, in the hematological DLBCL tumor model, Manara et al. clarified how Zotatifin has important synergy with a wide variety of ferroptosis inducers across multiple in vitro and in vivo studies. Furthermore they also observed a strong synergy also with Zotatifin combined with IFNγ, a cytotoxic T-cell effector cytokine known to promote ferroptosis in target cells. Based on this findings, they assessed that Zotatifin can sensitize tumors to CAR T-cell-mediated ferroptosis enhancing their anti-lymphoma activities [165].

#### NK cells

Several clinical trials using (autologous, allogeneic or modified) NK cells have shown promising results [166–168]. Despite these encouraging results, the immune suppressive tumor microenvironment activity can inhibit the activation and tumor infiltration of NK cells [169]. Kim et al. performed a study, in vitro and in vivo, to evaluate the synergistic combination effects of the ferroptosis inducer ferumoxytol and NK cell-mediated ferroptosis cancer therapy in prostate cancer model [170]. In particular, they used ferumoxytol for evaluating the contribution of cancer cell ferroptosis in NK cell therapy. Interestingly, the authors observed that the NK cells treatment, without ferumoxytol, showed a similar level of ferroptosis with ferumoxytol. These data suggest that the NK cells may induce the ferroptosis of cancer cells. Furthermore, they investigated the cancer cell killing efficacy of combined ferumoxytol and NK cell therapy. Interestingly, they showed that the co-treatment synergistically increased the cell lysis rate synergistically at higher levels compared to the monotreatment (NK cells or drug). Moreover, they observed that the co-treatment of NK cells and ferumoxytol-mediated ferroptosis significantly increased IFNγ secretion compared to NK cell treatment alone. The authors observed that the ferumoxytol mediates the ferroptosis of cancer cells by upregulating the NK cells by activating the surface molecules that finally drives to the cytotoxic effect of NK cells. In particular, they showed that ferumoxytol induced ferroptosis trough the NKG2D ligands (ULBP) in tumor cells. Upregulated NKG2D ligands in tumor cells can then activate the NK cells. Finally, the activated NK cells released an increased amounts of IFNγ. Another interesting result that they showed in their study was that the enhanced IFNγ observed in the treatment of NK cells + ferumoxytol mediated ferroptosis induced the PD-L1 upregulation in cancer cells. Hence, they added a PD-L1 inhibitor (aPD-L1) to the NK cells and ferumoxytol combination observing an increase in the ferroptosis-mediated tumor cell killing effect.

For in vivo evaluation, they performed intratumoral delivery of NK cell therapy generating a PC-3 xenograft mouse model in athymic nude mice. They then divided the mice into 4 groups (non-treated (DPBS), NK cell therapy, NK cell + ferumoxytol, and NK cell + ferumoxytol + aPD-L1). As expected, the NK cells + Ferumoxytol + aPD-L1 treatment showed a significant enhancement of T cell activation compared to other groups. Noteworthy, the addition of aPD-L1 therapy changed CD8^+^ T-cell profile. They observed that the cell ratio between CD8^+^ cytotoxic T-cell and Treg was significantly increased by the addition of aPD-L1 (NK cells + Ferumoxytol + aPD-L1).

### Side effects of ferroptosis inducers and immunotherapy combined treatment

Monitoring for potential side effects will be necessary because of the adverse effects observed in pre-clinical studies. In particular the accumulation of lipid peroxides can have deleterious effects on normal tissues and can lead to organ damage as in the liver and kidneys [171]. At the same time, inducing ferroptosis can lead to the death of stem cells and damage to the bone marrow, which may impact hematopoiesis and result in bone marrow suppression [69, 171]. It is also known that cytotoxic CD8^+^ T-cells, highly specialized in anti-tumor activity, can be affected by oxidized lipids accumulated upon ferroptosis inducers administration. Interestingly In addition, the GPX4 inhibitor RSL3 can impair the maturation and function of DCs and anti -tumor activity of NK cells [172, 173].

## Conclusions

The emerging role of ferroptosis in cancer treatment seems to be promising not only for the induction of cell death in cancer cells but for the potentiality to modulate anti-tumor activity in the TME. In particular its involvement in T-cells function could be an efficient tool to enhance the strength of check-point inhibitors and to improve CAR T-cells efficacy by favoring their killing activity and inducing cancer cell death. Ferroptosis role in support of NK cells administration is not yet well validated but seems to provide a favorable help to their efficacy. The limit that must be overcome is linked to balance between the pro- and anti-tumoral or anti-tumoral effect exerted by ferroptosis in the TME. This implies the selection of ferroptosis inducers able to stimulate immune cells with anti-tumor function and inhibiting those with pro-tumoral activity. The pre-clinical data on the combined therapy between ferroptosis inducers and immunotherapy will prompt the development of clinical trials aimed at verifying the efficacy of this new therapeutic approach in vivo, in particular in cancer patients resistant to conventional treatment. The use of compounds aimed to modulate ferroptosis is an innovative approach that can provide a new opportunity for cancer treatment, especially in case of relapse where current therapy is failing.

## Data Availability

No datasets were generated or analysed during the current study.

## Abbreviations

DC: Dendritic Cells
Treg: T regulatory cell
NK: Natural Killer
TAM: Tumor Associated Macrophages
ICI: Immune Check-point Inhibitor
CTL4: Cytotoxic T lymphocyte-associated antigen 4
PD-1: Programmed cell Death protein 1
PD-L1: PD-1 Ligand
CAR T-cell: Chimeric Antigen Receptor T cell
PARP-1: Poly(ADP-Ribose) Polymerase-1
AIF1: Apoptosis Inducing Factor 1
RIPK1: Receptor Interacting Protein Kinase 1
ROS: Reactive Oxygen Species
H_2_O_2_: Hydrogen peroxide
GPX4: Glutathione Peroxidase 4
Erastin: Eradicator of Ras and ST
Cys2: Cystine
Cys: Cysteine
Glu: Glutammate
GSH: Glutathione
RSL: RAS Selective Lethal
L-OOH: Lipid hydroperoxides
L-OH: Lipid alcohols
PUFA: Polyunsaturated Fatty Acids
4-HNE: 4-hydroxynonenal
PTM: Post-Translational Modifications
EGRQ: Early Growth Response 1
ACSL3: Acyl-CoA Synthetase Long Chain Family Member 3
HAT: Histone acethyltransferase
HDAC: Histone deacetylases
DNMT: C
m6A: N6-methyladenine
ncRNA: non-coding RNA
lncRNA: long non-coding RNA
miRNA: microRNA
IFNγ: Interferon γ
FSP1: Ferroptosis suppressor protein 1
TRIM: Tripartite motifs
TF: Transferrin
TFR1: Transferrin Receptor 1
UFM1: Ubiquitin fold modifier protein 1
NADH/NADPH: Nicotinamide Adenine Dinucleotide/Nicotinammide Adenina Dnucleotide Phosphate
DHODH: Dihydroorotate dehydrogenase
SKP2: S-phase kinase-associated protein 2
CCL: Chemokine ligand
PGE2: Prostaglandin E2
ZVI-NP: Zero-valent iron nanoparticles
DAMPs: Damage-associated molecular patterns
ICD: Immunological cell death
HMGB1: High mobility group box 1
HSP: Heat shock protein
MDA: Malondialdehyde
4-HNE: 4-hydroxynonenal
STAP3: Transmembrane ferrireductase
MDSC: Myeloid-Derived Suppressed Cell
TNBC: Triple Negative Breast Cancer
LAR: Luminal Androgen Receptor
AR: Androgen Receptor
PG: Phosphoglycerate
PGAM1: Phospho-Glycerate Mutase 1
HCC: Hepato-Cellular Carcinoma
ChOX: Cholesterol Oxidase
Fe-MOF: Iron Metal-Organic Framework
TXNRD: Pyridine Nucleotide Oxidoreductases
BET: Bromodomain and Extra Terminal domain)
AKR1C2: Aldo-Keto Reductase family 1 member C2
YTHDC2: YTH N6-Methyladenosine RNA Binding Protein C2
METTL3: N6-adenosine-methyltransferase complex
WTAP: WT1 Associated Protein
NRF2: Nuclear factor erythroid 2-Related Factor 2
FTO: Fat mass and obesity associated gene
IGF2BP: Insulin-like Growth Factor 2 mRNA-Binding Proteins
NMD: Nonsense-mediated mRNA decay
UPF1: Up-frameshift suppressor 1
USP8: Ubiquitin specific protease 8
SAS: Sulfasalazine
MEF: Mefloquine
FDA: Food and Drug Administration
LPCAT3: LysoPhosphatidylCholine AcylTransferase 3
PDT: Photodynamic therapy
LNP: Lipid nanoparticle
FAO: Fatty Acid Oxidation
CPT1A: Carnitine Palmitoyl Transferase 1A
ATMP: Advanced Therapy Medicinal Product
ROR1: Receptor tyrosine kinase-like Orphan Receptor 1
RTK: Receptor Tyrosine Kinase
NSCLC: Non-Small Cell Lung Cancer
DLBCL: Diffuse Large B-cell Lymphoma
